# Role of palliative care in centers performing maternal–fetal interventions

**DOI:** 10.3389/fped.2023.1223710

**Published:** 2023-07-07

**Authors:** Erin Rholl, Steven R. Leuthner

**Affiliations:** ^1^ Department of Pediatrics, Division of Neonatology, Medical College of Wisconsin, Milwaukee, WI, United States; ^2^ Children's Wisconsin, Milwaukee, WI, United States

**Keywords:** palliative care, fetal intervention, fetal surgery, ethics, shared decision-making, goals of care, quality of life, fetal center

## Abstract

Advancements in maternal–fetal interventions have allowed for direct fetal access, shifting the focus of interventions from maternal health for fetal health to a focus on sole fetal/neonatal benefit. Given that access to the fetus can only be obtained through the mother, there are ethical considerations important to consider when counseling the maternal–fetal dyad. The goals of maternal–fetal interventions range from improved fetal/neonatal survival to decreased long-term morbidities and improved quality of life. However, interventions to improve quality of life may not always achieve their desired result. Additionally, maternal–fetal interventions have risks such as premature birth and other complications that should be heavily considered as they may offset the potential benefits of the procedure. While some families elect for a maternal–fetal intervention, doing every potential postnatal intervention may not be in alignment with their goals depending on the outcome of the intervention. Given the complex, value-laden decision-making that is crucial to counseling parents about decisions surrounding maternal–fetal interventions and subsequent neonatal care, palliative care specialists should be utilized in fetal centers. Palliative care specialists are trained to assist with complex, goal concordant decision-making and can guide families and medical teams through the decision points that arise during the treatment journey.

## Introduction

Maternal–fetal interventions have advanced since their inception in the second half of the 20th century (1). More hospitals have developed fetal centers that bring together maternal–fetal medicine and pediatric specialists. Despite medical advancements, many maternal–fetal interventions remain in the realm of clinical innovation or research inspiring ethical dialogue about clinical practice (2). In this paper, we argue that inclusion of palliative care specialists is standard in fetal centers offering maternal–fetal interventions. We review the history of maternal–fetal interventions and how palliative care has evolved to support parents and teams in fetal centers. We use hypothetical cases to support our points.

### Evolution of fetal interventions

While maternal–fetal interventions are often discussed in the context of surgical interventions, interventions to the maternal–fetal dyad started well before direct surgical access to the fetus (3). Steady advancements in obstetric diagnostics led to earlier detection of abnormalities in fetal development such as congenital anomalies. With these advancements, interventions shifted from those with goals of maternal health to maternal health for fetal health to direct fetal benefit (2). Interventions focused on maternal health for fetal health include glycemic control for diabetes. There is a low maternal risk, and the maternal–fetal dyad health will improve. Interventions for sole fetal/neonatal benefit include indirect and direct access to the fetus. Interventions with indirect access include those with a lower risk to the mother, steroids for fetal lung maturity, to those with moderate risk, digoxin for fetal arrhythmia. Direct interventions are often surgical. Surgical maternal–fetal intervention techniques range from hysterotomy (access to the fetus from the exposed uterus) to fetoscopic (laparoscopic technique) to fetal image-guided surgery (FIGS) (guided by ultrasound).

**Figure 1:**
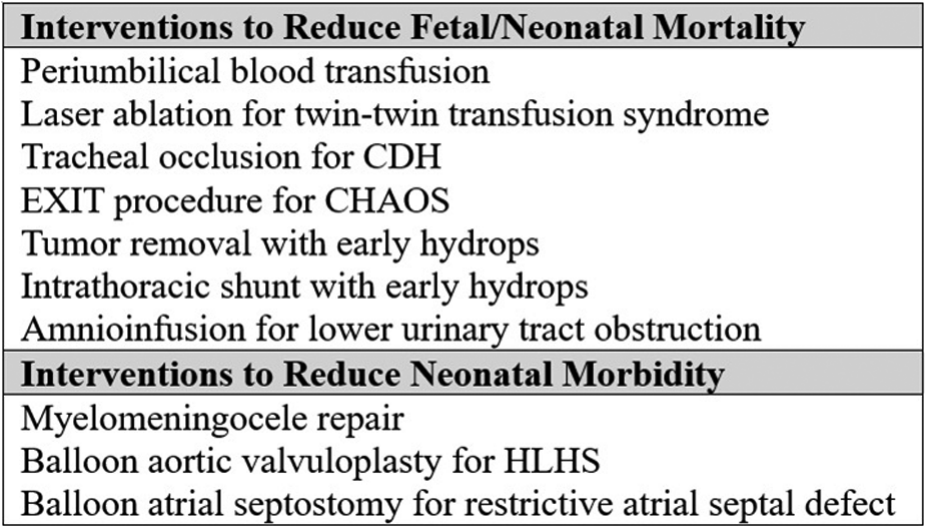
Fetal interventions categorized by goals.

Surgical maternal–fetal intervention goals have also evolved. Initially, the goals were to decrease fetal/neonatal mortality when death was anticipated. Maternal–fetal intervention success led to the ability to perform interventions to decrease morbidities in cases where the fetus would be expected to survive. Figure 1 provides examples of maternal–fetal surgical interventions classified by goals. More recent techniques have led to less maternally invasive fetoscopic and FIGS approaches. This is done to reduce maternal surgical risk and fetal prematurity risk. An example of this transition is congenital diaphragmatic hernia (CDH), which was initially approached by hysterotomy. The current approach for CDH is fetoscopic endoluminal tracheal occlusion (FETO) as noted in the tracheal occlusion to accelerate lung growth trial (TOTAL) (4, 5). The trial reported benefits to neonates with severe CDH. However, any intervention, even minimally invasive, still carries risks to the mother and fetus such as pregnancy loss or preterm labor. While the TOTAL study reported improvement in survival, time to repair, and decreased ECMO, more than half of FETO patients did not survive and more were born prematurely (4, 5). In some cases, a hysterotomy may still be performed such as if an occlusion device is not removed prenatally.

Even if a maternal–fetal intervention is performed without complications, the desired outcome may not be achieved or be no better than cases receiving standard care, meaning downstream complex decision-making will still occur. CDH remains an example of this as postnatal neonatal care has advanced, and several centers are reporting improved and comparable survival outcomes of severe CDH comparable to the TOTAL trial (6, 7). Regardless of the outcome, families offered maternal–fetal interventions will experience cycles of grief along their journey.

In summary, while there have been advances in maternal–fetal interventions and fetal care, there are cases that do not meet intervention criteria, potential complications from intervention leading to prematurity or added morbidities or death, times the intervention might be technically successful but not have the desired effect, or even with success now lead to a chronic medical condition requiring neonatal therapies. All these situations require complex understanding and medical decision-making that are ongoing in the care of these infants, which is what palliative care consultation offers. While these are reasons for palliative care in fetal centers, there are still misconceptions about palliative care. To address these, we review the history of perinatal palliative care next.

### History of palliative care

Palliative care concepts have been involved in the prenatal space since the 1990s (8). Historically, palliative care was consulted for cases of aneuploidy or severe anomalies as an alternative to pregnancy termination when comfort care was the only option given (8). Perinatal palliative care programs evolved to support families receiving a range of diagnoses that paralleled conversations in neonatal care. This included diseases considered fatal or life-limiting to those associated with a high degree of morbidity and mortality to assist with decision-making upstream (9). This process developed as the WHO recognized that palliative care should start early in the disease process (10). From there, the practice changed by recognizing that early could mean prenatally. Despite the evolution of palliative care, many providers still incorrectly assume that palliative care is synonymous with comfort care or hospice. Palliative care is now involved in cases where fetal or neonatal interventions are pursued, partnering with families and other medical specialists to ensure care remains goal concordant and assisting in complex symptom management. Palliative care may also serve as a bridge to outpatient hospice for families that do not want interventions or that desire comfort and quality-of-life-focused support while also pursuing interventions. Adult patients must decide between either a curative or intensive medical intervention or a comfort-focused, hospice approach. One struggle families of pediatric patients previously faced was how to reconcile the desire for interventions while also recognizing how hospice models of care may benefit knowing their child has a life-limiting condition. After the Affordable Care Act passed, Section 2302 Concurrent Care for Children allowed for curative or life-prolonging interventions in conjunction with hospice for children with Medicaid (11). Clearly, palliative care has evolved since initially presented as an alternative to pregnancy termination. David Munson proposes pediatric palliative care involvement for three main types of fetal/neonatal cases: (1) fetal or immediate postnatal surgery is intended to save the infant's life but carries its own risk of morbidity or mortality, (2) surgery may extend life but another context should be considered, and (3) interventions focused on improving quality of life (12).

Given the complexity and uncertainty integrated with the maternal–fetal space, the goals of the expectant mother and family should drive treatment recommendations and plans. Palliative care providers note that early involvement helps them better understand family goals to assist with decision-making more effectively throughout a patient's medical journey. For families receiving a diagnosis where a maternal–fetal or neonatal intervention may be offered, early means at the fetal diagnosis and prior to the decision to pursue an intervention. Palliative care can help inform and support the alternative options of either termination or comfort care if that meets the family's goals, as well as interventions. Some may ask why might palliative care be useful in cases of more aggressive maternal–fetal interventions? Palliative care providers are trained to assist families in formulating goals of care and complex decision-making while providing longitudinal support throughout the illness trajectory. They review tradeoffs and risks anticipated with all potential paths for all parties, helping families figure out which risks they can accept and which might cause the most regret. For families desiring a fetal or postnatal intervention, palliative care can remain involved in determining the specific goals of an intervention and assessing whether the goals have been achieved after the intervention (13). If the treatment plan is no longer meeting a family's goals the palliative team can assist in discussions surrounding redirection of care or reassessment of goals. While most studies reviewing the benefits of perinatal palliative care have focused on lethal conditions, studies on pediatric cardiology have shown benefits in family-reported outcomes with upstream palliative care in the prenatal and neonatal period for families whose infants received a diagnosis of congenital heart disease even for families desiring interventions (14, 15).

Palliative care can also help balance the ethical concerns in fetal medicine. The maternal–fetal dyad is unique as maternal–fetal surgical interventions require direct action through the mother to access the fetus. The American Academy of Pediatrics (AAP) and American College of Obstetricians and Gynecologists (ACOG) have statements guiding clinicians at fetal centers ensuring risks to the pregnant woman are not overlooked when focusing on potential fetal benefits (16–18). The very nature of a fetal center can unintentionally be balanced toward fetal benefit as websites and marketing often overemphasize benefits to the fetus and underemphasize potential maternal procedural risks. One survey of maternal–fetal medicine, neonatology, and pediatric surgery providers noted that most centers considered the neonatal benefit in the highest regard with maternal risk being the second (19). Neonatologists were more fetocentric, surgeons were more risk-sensitive, and maternal–fetal medicine was more focused on maternal autonomy and family impact (19). Great care must be taken to ensure that families are adequately counseled on all reasonable options and not inadvertently pressured into a plan not in alignment with their goals.

We will use several hypothetical cases to further illustrate how palliative care involvement is beneficial for cases where maternal–fetal interventions are considered.

### Case examples

#### Case 1: intervention to prevent neonatal mortality–congenital high airway obstruction syndrome

Maeve is a 25-year-old G3P0 woman pregnant with a fetus that at 21 weeks was diagnosed with congenital high airway obstruction syndrome (CHAOS) and is referred to a fetal center. She meets with several specialists that counsel on the risks that include death without intervention as well as long-term tracheostomy dependence (20). The team offers to perform an EXIT procedure to reduce the risk of asphyxia and increase her infant's chance of survival.

Risks to the pregnant woman, in this case, include undergoing an EXIT procedure, which carries risks related to the operation and future pregnancies. A mother who is a gravida 1 that desires future pregnancies may consider those risks more significant than a mother who has other children and is not planning on conceiving after the current pregnancy. Does the fetus have signs of hydrops which increase the risk of mortality even with intervention (20)? Palliative care specialists are trained in eliciting values, worries, regrets, and hopes. In cases where there is no easy or good path forward, palliative care providers explore those perspectives with parents to help them understand which path might be best for them.

Palliative care meets with Maeve and her partner to discuss options. They had two prior miscarriages and are motivated to give their baby a chance of survival. While they acknowledge there are risks to Maeve and her future fertility, they would still like to proceed with the EXIT procedure if her infant does not develop hydrops. If their infant survives, the risks of tracheostomy, ventilator, and enteral tube feeding dependence are acceptable if their infant can communicate even if not verbally. Maeve's pregnancy continues without issue, and she undergoes the EXIT procedure. A tracheostomy was placed during the procedure and her infant, Taylor, was born. He is now 3 years old, remains tracheostomy dependent and nonverbal, and communicates through sign language. Palliative care has continued to follow the family as there are future procedures anticipated.

#### Case 2: intervention to decrease neonatal morbidity–myelomeningocele

Angela is a 34-year-old G1P0 woman pregnant with a fetus that at 19 weeks is diagnosed with L1–L2 myelomeningocele. After counseling with her maternal–fetal medicine physician, Angela was transferred to a center performing fetoscopic myelomeningocele repairs. The fetal team offered to do the surgery at 22 weeks. After meeting with the maternal–fetal surgeon, neurologist and neurosurgeon Angela met with palliative care.

The palliative care physician supported Angela in her grief over the loss of a normal pregnancy. They reviewed what Angela learned about the diagnosis as well as available options. In particular, they explore Angela's goals for her child, or what is important? Is it most important that her child be born alive and she can care for her? Or is it most important she does everything to reduce disability? Because undergoing this procedure puts her baby at risk of death the baby otherwise would not have to take. Angela reports that with standard postnatal care, her infant is expected to be wheelchair dependent, require a cerebrospinal fluid shunt, and have difficulty controlling going to the bathroom. The fetal team told her with prenatal repair, they hope to improve functional skills and potentially avoid a shunt (21, 22). Angela's main concern is maximizing future functional skills. She is willing to accept potential maternal and fetal risks of the procedure including the low but irreversible risk of premature delivery which depending on how premature could lead to more significant morbidities. The palliative physician explored the potential developmental outcomes of a premature infant with myelomeningocele as each week of gestation progressed past the procedure date of 22 weeks. Angela decided to choose comfort care if her baby was born prior to 25 weeks based on her acceptable thresholds for quality of life (23).

Angela underwent the procedure with the palliative care provider available in case her infant had to be delivered. The procedure was successful, and Angela remains hospitalized for monitoring. Unfortunately, at 23 weeks, Angela goes into preterm labor and her infant, Jaxon, needs to be delivered. Given the prior birth plan, the obstetrical team, labor and delivery staff, and palliative team quickly support Angela with a delivery that is safest for her and a comfort care plan until Jaxon's death, which is consistent with her initial goals of wanting to reduce disability. The palliative care team remains involved in Angela's care for bereavement support for the next year.

#### Case 3: intervention to decrease neonatal morbidity–critical aortic stenosis

Monique is a 26-year-old G3P2 who at 20 weeks was informed her fetus had critical aortic stenosis. Monique was referred to a fetal center where the pediatric cardiologist counseled that her fetus was at high risk to progress to hypoplastic left heart syndrome (HLHS) discussing the risks and morbidities associated with single ventricle pathway and transplant. Monique was offered the option of prenatal aortic valvuloplasty.

Monique meets with palliative care at the center to review what she learned from the other specialists. Monique expressed her worries about the morbidities of single ventricle physiology and interest in the intervention to prevent progression to HLHS. Palliative care assists in discussions with Monique and cardiology about the potential outcomes of the intervention. First, if the fetus died during the intervention, palliative care would help with bereavement support. Second, while low risk, what would be the plan if the procedure triggered preterm labor? The teams discussed gestational age and weight thresholds below which they would not perform any cardiac interventions. Palliative care discussed the potential for Monique's infant to still progress to single ventricle physiology after the intervention explaining that comfort care and trial of surgical interventions were both still options available.

Monique underwent the maternal–fetal intervention and completed the rest of her pregnancy without complication. Her infant, Sophia, was born at 39 weeks. Unfortunately, postnatal assessment determined that Sophia progressed to HLHS and she was not a candidate for biventricular repair and cardiology recommended the single ventricle pathway. The palliative physician reviewed Monique's goals for Sophia, discussing options ranging from comfort care or starting with the first surgery and assessing how Sophia tolerates the single ventricle pathway. In reviewing Monique's goals, they acknowledged the reason she underwent the maternal–fetal intervention accepting the risk of fetal death was because Monique felt life with a single ventricle would be too burdensome. To begin the single ventricle process now, might mean that goals changed with bonding. From the beginning of the intervention process, it is important to discuss if the plan to “do everything” is based on quality vs. life overall. Parents should be allowed to change their goals as they learn more about their children and their condition. However, we as providers should remain ethically consistent and understand that we cannot allow a mother to undergo a fetal intervention to avoid the single ventricle pathway with a small but real risk of fetal death and then not allow comfort care postnatally for parents' desiring that path.

Monique, having bonded with Sophia, wanted to give her a chance for more time and elected to pursue surgical interventions. While goals changed, Monique still valued quality of life and appreciated that comfort care remained an option. Sophia's course had complications and cardiology began discussions about transplant after Sophia's first operation. Palliative care remained involved in Sophia's care and was part of the transplant evaluation. On review of Sophia's journey, her current medical supports, benefits, and burdens of a transplant Monique decided that transitioning to comfort care was most in alignment with her goals. Palliative care and cardiology discussed the logistics of that transition and arranged home hospice support for Sophia.

Now let's consider an alternative outcome in the case, Monique elects to pursue a transplant for Sophia. Sophia survives the transplant process and after an uneventful year after the transplant palliative care is no longer actively following. When Sophia becomes a teenager, palliative care is re-engaged. Palliative care helps Sophia fill out the “Voicing My Choices” advanced care planning guide that allows Sophia to become an active participant in discussions about her care (24).

**Figure 2:**
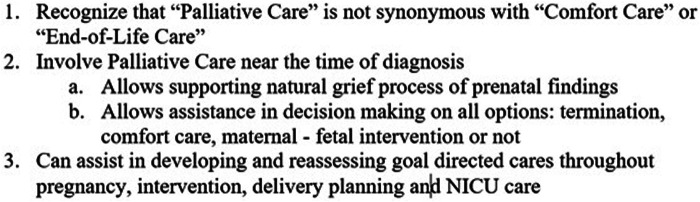
Pearls for involving perinatal palliative care.

These three cases provide only some examples of how working with a palliative care team can provide support for families and physicians in addressing goal-directed care in maternal, fetal, and neonatal management decisions that start with a fetal diagnosis. They demonstrate that palliative care teams can support termination, comfort care approaches, or fetal interventions that lead to neonatal interventions, and how early involvement provides added support in parental decision-making later (Figure 2). Centers interested in incorporating palliative care services in their fetal/prenatal services should start by performing a needs assessment and stakeholder support to determine the best way to incorporate services locally. Education of all perinatal and pediatric subspecialty providers to recognize that “palliative care” and “comfort care” are not synonymous terms is critical to improved utilization. As palliative care teams become increasingly utilized in fetal centers, researchers should focus on parameters for active involvement. Future research on parent satisfaction and quality indicators of fulfilling tier goals would provide value for continued growth.

## Conclusion

Maternal–fetal interventions focusing solely on fetal or neonatal benefit can be expected to increase as interest grows with medical advancements. While less invasive techniques are being utilized, there remain maternal and fetal risks and the goal of the intervention is not always achieved. Even with successful maternal–fetal interventions, patients may continue to have long-term morbidities making them appropriate candidates for palliative care consultation. Incorporating palliative care into fetal centers should be standard to ensure balanced, goal-oriented discussions about the care plans and ensure longitudinal support for these maternal–infant dyads.

## Data Availability

The original contributions presented in the study are included in the article, further inquiries can be directed to the corresponding author.
